# Challenges in perioperative code status management: a national survey among Swiss anaesthetists

**DOI:** 10.1016/j.resplu.2025.101174

**Published:** 2025-11-22

**Authors:** Samuel K. Zumbrunn, Flavio Gössi, Benjamin Bissmann, Armon Arpagaus, Sebastian Gross, Christoph Becker, Simon A. Amacher, Raoul Sutter, Kai Tisljar, Ariane Rossi, Marc Lüthy, Luzius A. Steiner, Thierry Girard, Sabina Hunziker

**Affiliations:** a Medical Communication and Psychosomatic Medicine, University Hospital Basel, Basel, Switzerland; b Medical Outpatient Department, University Hospital Basel, Basel, Switzerland; c Faculty of Medicine, University of Basel, Basel, Switzerland; d Division of Internal Medicine, University Hospital Basel, Basel, Switzerland; e Department of Anaesthesiology and Intensive Care Medicine, Medical Center-University of Freiburg, Faculty of Medicine, University of Freiburg, Freiburg im Breisgau, Germany; f Intensive Care Unit, Department of Acute Medicine, University Hospital Basel, Basel, Switzerland; g Clinic for Anaesthesia, Intermediate Care, Prehospital Emergency Medicine and Pain Therapy, University Hospital Basel and University of Basel, Basel, Switzerland; h Sanität, Rettung Basel-Stadt, Justiz- und Sicherheitsdepartement des Kantons Basel-Stadt, Basel, Switzerland; i Post-Intensive Care Clinic, University Hospital Basel, Basel, Switzerland

**Keywords:** Anaesthesia, Code status discussion, DNR, Perioperative period, Resuscitation

## Abstract

**Aim:**

To evaluate current practices and beliefs among anaesthetists regarding perioperative code status discussions and management in surgical patients.

**Methods:**

A nationwide web-based survey was conducted in Switzerland among anaesthetists providers. Primary endpoint: proportion of participants routinely discussing code status with medium-risk patients based on the American Society of Anesthesiologists (ASA) physical status classification system (ASA 3–4). Secondary endpoints: proportion of code status discussions in low-risk (ASA 1–2) and high-risk (ASA 5) patients; perioperative intensive care unit (ICU) admissions, severe complications, therapy limitations and management of patients with Do Not Resuscitate (DNR) orders.

**Results:**

Of 496 respondents, 474 were included. 56.8 % (*n* = 269) of anaesthetists reported routinely discussing code status with medium-risk patients (ASA 3–4). Factors associated with routine code status discussion included greater experience (mean [±SD] 20.7 [±10.6] vs. 17.4 [10.8] years, adjusted OR 1.03 [95 % CI 1.02–1.05], *p* < 0.001), more exposure to cardiopulmonary resuscitation (CPR) (≥50 CPRs: 169/269 [62.8 %] vs. 93/205 [45.4 %]; adjusted OR 2.99 [95 % CI 1.39–6.42], *p* = 0.005), and awareness of institutional teaching (48/243 [19.8 %] vs. 13/180 [7.2 %], adjusted OR 2.93 [95 % CI 1.51–5.68], *p* = 0.001), though only 14.4 % reported such training. 76.3 % (*n* = 318) of anaesthetists reported discussing code status preoperatively with patients who have DNR orders. While 70.4 % (*n* = 285) supported respecting DNR orders perioperatively, 21.3 % (*n* = 86) felt they should not apply.

**Conclusion:**

Code status discussions are commonly reported for medium-risk patients, but formal education is limited. Experience and clinical exposure increase the likelihood of such discussions. Enhanced training and clearer guidelines are needed to support consistent ethical management of code status in the perioperative setting.

## Introduction

Survival to discharge of in-hospital cardiac arrest (IHCA) ranges from 15 % to 34 %, and up to 28 % of survivors suffer from neurological sequelae.^1^, ^2^, ^3^, ^4^ However, perioperative cardiac arrests (POCA), defined as cardiac arrests occurring between arrival in the operating area to 24 h postoperatively, differs from IHCA both in aetiology and management.^5^ While POCA survival is overall higher (31.7–34.5 %), neurological deficits still affect about one-third of survivors.^6^, ^7^

The incidence of cardiac arrests varies by setting. IHCA occurs in approximately 15–28 per 10,000 hospital admissions^1^ while POCA incidence ranges from 1 in 33 patients undergoing cardiac surgery^8^ to 0.2–1.1 per 10,000 adults undergoing non-cardiac surgery.^9^

Although the technical execution of cardiopulmonary resuscitation (CPR) in perioperative and in-hospital (IHCA) settings is similar, the context differs significantly. POCA often results from procedural complications or anaesthesia-related events. Continuous monitoring and immediate availability of trained personnel enable timely intervention, which could contribute to better outcomes.^5^ Known risk factors for POCA include preexisting heart failure, peripheral vascular disease, pulmonary circulation disorders, end-stage renal disease, electrolyte disorders, and cancer.^8^, ^10^, ^11^ Surgical patients are commonly classified using the American Society of Anesthesiologists (ASA) physical status classification system, which grades a patient’s overall preoperative health to estimate perioperative risk. The ASA system ranges from ASA 1, a healthy individual without systemic disease, to ASA 4, a patient with severe disease posing a constant threat to life; ASA 5 designates a moribund patient not expected to survive without surgery.^11^, ^12^, ^13^

Preoperative discussions about resuscitation preferences are recommended by the Swiss Academy of Medical Sciences^14^ and international guidelines.^15^ Notably, the committee on ethics of the American Society of Anesthesiologists recommends a process of required reconsideration for existing Do Not Resuscitate (DNR) orders before surgery, ensuring that treatment options and limitations are reviewed with the patient or next of kin in case a perioperative arrest occurs.^16^

However, previous research has shown that conversations about code status often are neglected or held inadequately and may result in decisions violating patients’ values or good clinical practice.^17^, ^18^ Moreover, both patients and health care providers often overestimate survival chances following cardiac arrest, potentially influencing resuscitation decisions.^17^, ^19^

Despite these recommendations, shared decision-making is often lacking. Surveys indicate that many surgeons and anaesthetists may not always follow DNR orders during procedures.^20^, ^21^ Up to 75 % of surgeons and 54 % of anaesthetists perceived them as perioperatively irrelevant in a recent study, and 18 % of anaesthetists and 38 % of surgeons routinely override them without involving the patient.^20^

Similarly, a recent survey of American gastroenterologists showed that the majority routinely reversed DNR orders, over 70 % skipped pre-procedure code status discussions, and only ∼20 % checked for an existing DNR order.^22^

No comprehensive Swiss data exist on perioperative code status management; this study aimed to assess anaesthetists’ practices and beliefs on the topic.

## Methods

### Participants and survey administration

Data were collected from February 20 to April 28, 2025, via a nationwide anonymous, multicentre, online survey of Swiss anaesthetists. The Swiss Society for Anaesthesiology and Perioperative Medicine (SSAPM) endorsed the survey and distributed the link. Teaching hospitals were contacted individually to identify anaesthetists who are not members of SSAPM. Department heads from the national registry of certified Swiss medical centres^23^ were also invited to share the survey link. Data were collected using REDCap,^24^, ^25^ which is compliant with the Swiss Data Protection Act (DSG) and the European General Data Protection Regulation (GDPR). Inclusion required active hospital practice and full survey completion.

### Ethics

Participation was voluntary. Participants were informed about the study’s objectives, assured confidentiality, and consent was implied by survey response. The ethics committee of northern and central Switzerland waived the need for formal ethical approval (Req-2024-01571).

### Training context in Switzerland

In Switzerland, postgraduate training in anaesthesiology is a 5-year national programme that includes 3–4.5 years of anaesthesiology and 6–24 months of intensive care medicine. Competencies in communication, ethics, resuscitation, and perioperative care are mandatory, with structured teaching and supervised consultations required. The final qualification consists of a written European Society of Anaesthesiology and Intensive Care (ESAIC) examination and an oral examination in Switzerland based on clinical case discussions.

### Questionnaire development

The survey was conducted and reported in accordance with the Checklist for Reporting Results of Internet E-Surveys (CHERRIES) guidelines^26^ and best practices for survey research^27^ guidelines.

It was developed in a three-step process: First, an initial draft was developed by the research team, consisting of three attending physicians (SH, FG, AA) and two residents (SZ, BB), based on existing literature and clinical expertise. Second, face validity was assessed by four senior anaesthetists (TG, LS, ML, SA), whose feedback was incorporated into the revised version of the survey. Third, the questionnaire was implemented in REDCap and pilot tested within the research team and senior anaesthetists to evaluate functionality, comprehension of instructions, and estimated completion time of 8 min. Final content validation was performed concurrently by the senior anaesthetists, resulting in minor adjustments before dissemination (Supplement 4). A bilingual native speaker (AR) translated the survey from German to French, both of which are official languages in Switzerland. Respondents were able to review and change their answers throughout the entire survey before final submission.

### Outcome measures

The **primary outcome** was the proportion of anaesthetists actively discussing the code status with their patients preoperatively on a regular basis, assessed among patients in the medium-risk group (ASA 3–4) based on the ASA classification.^13^, ^28^

The ASA classification categorizes patients according to their pre-anaesthesia comorbidities as follows: healthy (ASA 1), mild systemic disease (ASA 2), severe systemic disease (ASA 3), severe systemic disease that is a constant threat to life (ASA 4), moribund and not expected to survive without surgery (ASA 5), and brain-dead (ASA 6).

**Secondary endpoints** were^1^ the proportion of anaesthetists actively discussing the code status in (a) low- (ASA 1–2) and (b) high-risk (ASA 5) patients,^2^ proportion of anaesthetists discussing perioperative intensive care unit (ICU) admission, severe complications and^3^ postoperative therapy limitations in three previously described ASA-based risk groups,^4^ proportion of anaesthetists discussing the perioperative code status with patients in case of a documented DNR order. Further secondary endpoints were (5) anaesthetists’ perceptions about perioperative code status, DNR orders and perioperative CPR.

To ensure consistent analysis, outcome variables were dichotomized according to the distribution of responses: for the primary endpoint and secondary endpoints,^1^, ^2^, ^3^ responses of ‘almost always’ or ‘often’ were categorized as ‘discussion yes’, while ‘rarely’ or ‘almost never’ were classified as ‘discussion no’. For the remaining secondary endpoints,^4^ responses of ‘yes’ or ‘rather yes’ were grouped as ‘discussion yes’, and ‘rather no’ or ‘no’ as ‘discussion no’.

### Baseline characteristics and associated factors

Baseline characteristics included age, self-reported gender, hospital size, function (i.e. resident, attending physician), specialization (i.e. anaesthesiology, internal medicine, intensive care medicine), and years of clinical experience.

The following physician-related factors potentially associated with outcomes were assessed: (i) number of premedication consultations per week, (ii) number of anaesthesia procedures performed per week, (iii) number of perioperative and (iv) overall resuscitations attended, (v) estimated survival rate with independence in activities of daily living following POCA. Further, anaesthetists’ individual preferences (summarized in Table 1b), were included as associated factors. Further, the following patient-related factors were assessed: (i) advanced age (e.g. >80 years), (ii) young age (e.g. <50 years), (iii) elective admission, (iv) inpatient status, (v) high expected perioperative complication risk, (vi) low expected perioperative complications risk, (vii) pre-existing complete need for care (e.g. nursing home, home care), (viii) low expected quality of life after surgery, (ix) significant comorbidities (e.g. advanced cardiac or pulmonary disease), (x) high iatrogenic risk of circulatory arrest (heart surgery, intrathoracic procedures), (xi) cognitive impairment or dementia, (xii) presence of a documented advance directive.

Baseline variables and associated factors were dichotomized to allow for more precise analysis. Patient-related factors rated as ‘not necessary’ or ‘often not necessary’ were grouped into the ‘no’ category. Ratings of ‘rarely necessary’, ‘sometimes necessary’, ‘often necessary’, or ‘always necessary’ were grouped into the ‘yes’ category.

Physician-related factors were grouped into ‘agreement’ (‘tend to agree’, ‘agree’, ‘absolutely agree’) and ‘disagreement’ (‘rather disagree’, ‘disagree’, ‘absolutely disagree’) to reflect general attitudes toward code status discussions. The full questionnaire is available in Supplement 4.

### Statistical analysis

We used descriptive statistics to summarize and present the data. Baseline characteristics and outcomes were stratified according to primary and secondary endpoints and groups were compared using Pearson’s *χ*^2^-Test and the two-tailed Student’s *t*-test or Wilcoxon rank-sum test, as appropriate, following an assessment of data distribution. Categorical variables were reported as proportions (percentages) and continuous variables as means with standard deviations. We also calculated univariable and age-/gender-adjusted logistic regressions models to assess associations. Significance was set at *p* < 0.05. Analyses used STATA 15.0 (Stata Corp., College Station, TX, USA).

## Results

### Baseline characteristics

Of 496 physicians participating in the survey, 22 (4.4 %) were excluded due to missing responses to the primary endpoint. Among the 474 physicians included in the final analysis, the mean age (±SD) was 46.4 (±11.1) years and 202 (42.6 %) were female. Most participants were consultants 152 (32.5 %) or attending physicians 144 (30.4 %). Average clinical experience was (mean [±SD]) 19.2 [±10.8] years and 392 (82.7%) were board certified. A total of 194 (40.9 %) worked in a centre hospital and 262 (55.3 %) had CPR experience exceeding 50 cases. On average, participants reported performing (mean [±SD]) 11.4 [±9.1] premedication consultations and 20.2 [±13.9] anaesthetic procedures per week (Table 1a).

**Table 1a:** Anaesthetists’ related factors conducting perioperative code status discussions.

**Factor**	**Categories**	***N***	**All**	**Discussion** **no**	**Discussion** **yes**	**Univariable OR (95 % CI)**	***p*-value**	Multivariable OR (95 % CI)*	***p*-value**
***Demographic factors of clinicians***									
***N***		474	474	205	269				
**Age (years), mean (SD)**		474	46.4 (11.1)	44.6 (11.3)	47.8 (10.7)	1.03 (1.01, 1.04)	0.002	1.03 (1.01, 1.05)	<0.001
**Gender, *n* (%)**	**Female**	474	202 (42.6 %)	72 (35.1 %)	130 (48.3 %)	1.73 (1.19, 2.51)	0.004	1.94 (1.32, 2.84)	0.001
**Hospital size, *n* (%)**	**Centre hospital with maximum care (e.g. university hospital)**	474	194 (40.9 %)	84 (41.0 %)	110 (40.9 %)	(Ref.)		(Ref.)	
	**Cantonal hospital**		123 (25.9 %)	54 (26.3 %)	69 (25.7 %)	0.96 (0.62, 1.54)	0.92	0.78 (0.49, 1.26)	0.31
	**Regional hospital**		91 (19.2 %)	38 (18.5 %)	53 (19.7 %)	1.06 (0.64, 1.76)	0.81	0.68 (0.39, 1.19)	0.18
	**Private practice**		66 (13.9 %)	29 (14.1 %)	37 (13.8 %)	0.97 (0.56, 1.71)	0.93	0.59 (0.31, 1.11)	0.1
**Function, *n* (%)**	**Resident**	474	97 (20.5 %)	56 (27.3 %)	41 (15.2 %)	(Ref.)		(Ref.)	
	**Attending physician**		144 (30.4 %)	62 (30.2 %)	82 (30.5 %)	1.80 (95% CI 1.07, 3.04)	0.026	1.77 (1.04, 2.99)**	0.035
	**Consultant**		154 (32.5 %)	58 (28.3 %)	96 (35.7 %)	2.26 (1.34, 3.79)	0.002	2.48 (1.46, 4.21)**	0.001
	**Head physician**		79 (16.7 %)	29 (14.1 %)	50 (18.6 %)	2.35 (1.28, 4.33)	0.006	2.86 (1.53, 5.37)**	0.001
**Specialty title, yes, *n* (%)**		474	392 (82.7 %)	160 (78.0 %)	232 (86.2 %)	1.76 (1.09, 2.85)	0.02	1.98 (1.21, 3.24)**	0.006
**Clinical experience in years, mean, SD**		474	19.2 (10.8)	17.4 (10.8)	20.7 (10.6)	1.03 (1.01, 1.05)	0.001	1.03 (1.02, 1.05)**	0.001
**Physician related factors**									
**Premedication consultations, mean, SD**		474	11.4 (9.1)	11.4 (8.5)	11.4 (9.5)	1 (0.98, 1.02)	0.991	1 (0.98, 1.02)	0.853
**Anaesthesia procedures per week, mean, SD**		474	20.2 (13.9)	20.5 (16.8)	19.9 (11.3)	1 (0.98, 1.01)	0.675	1 (0.98, 1.01)	0.699
**Number of performed perioperative CPR, *n* (%)**	**Below 10**	474	281 (59.3 %)	138 (67.3 %)	143 (53.2 %)	(Ref.)		(Ref.)	
	**10–50**		170 (35.9 %)	61 (29.8 %)	109 (40.5 %)	1.72 (1.1.7, 2.56)	0.006	1.65 (1.07, 2.54)	0.024
	**Over 50**		23 (4.9 %)	6 (2.9 %)	17 (6.3 %)	2.73 (1.05, 7.14)	0.04	2.41 (0.89, 6.53)	0.084
**Number of performed CPR in general, *n* (%)**	**Below 10**	474	39 (8.2 %)	24 (11.7 %)	15 (5.6 %)	(Ref.)		(Ref.)	
	**10–50**		173 (36.5 %)	88 (42.9 %)	85 (31.6 %)	1.54 (0.76, 3.14)	0.23	1.49 (0.71, 3.14)	0.29
	**Over 50**		262 (55.3 %)	93 (45.4 %)	169 (62.8 %)	2.91 (1.45, 5.81)	0.003	2.99 (1.39, 6.42)	0.005
**Estimated survival rate after perioperative CPR, mean (SD)**		420	40.04 (22.32)	40.3 (21.8)	39.8 (22.7)	1 (0.99, 1.01)	0.822	1 (0.99, 1.01)	0.66

***Factors related to the perioperative discussion***									
**Surgeon responsible for code status discussions yes, *n* (%)**	**Anaesthetist**	451	53 (11.8 %)	17 (8.7 %)	36 (14.1 %)	(Ref.)		(Ref.)	
	**Both Anaesthetist and Surgeon**		381 (84.5 %)	163 (83.6 %)	218 (85.2 %)	0.63 (0.34, 1.16)	0.14	0.56 (0.30, 1.06)	0.074
	**Surgeon**		17 (3.8 %)	15 (7.7 %)	2 (0.8 %)	0.063 (0.013, 0.31)	0.001	0.06 (0.012, 0.29)	0.001
**Advance directives (AD) assessed prior to intervention, *n* (%)**		422	234 (55.5 %)	60 (33.3 %)	174 (71.9 %)	5.12 (3.37, 7.77)	<0.001	5.33 (3.46, 8.21)	<0.001
**Are prior AD relevant in a perioperative phase, yes, *n* (%)**		423	299 (70.7 %)	120 (66.7 %)	179 (73.7 %)	1.4 (0.92, 2.13)	0.119	1.34 (0.87, 2.07)	0.184
**Decisional preference in perioperative CSD, *n* (%)**	**Physician**	448	28 (6.2 %)	12 (6.2 %)	16 (6.2 %)	(Ref.)		(Ref.)	
	**Physician/patient**		346 (77.2 %)	157 (81.8 %)	189 (73.8 %)	0.90 (0.41, 1.96)	0.79	0.92 (0.42, 2.03)	0.83
	**Patient**		74 (16.5 %)	23 (12.0 %)	51 (19.9 %)	1.66 (068, 4.07)	0.26	1.49 (0.59, 3.72)	0.39
**Teaching for residents, yes, *n* (%)**		423	61 (14.4 %)	13 (7.2 %)	48 (19.8 %)	3.16 (1.66, 6.04)	<0.001	2.93 (1.51, 5.68)	0.001
**Do you think perioperative CSD scare the patient, yes, *n* (%)**		423	229 (54.1 %)	113 (62.8 %)	116 (47.7 %)	0.54 (0.37, 0.8)	0.002	0.53 (0.35, 0.79)	0.002

Data presented as *n* (%) or mean (SD) unless otherwise specified. Abbreviations: **OR,** Odds Ratio; **SD**, standard deviation; **n**, number; **CPR** cardio-pulmonary resuscitation; **AD** advance directive; **CSD** Code Status Discussion.

* Adjusted for age, gender.

** Adjusted for gender only due to collinearity with age.

**Table 1b:** Patient related factors and anaesthetists’ attitudes in perioperative code status discussions.

**Factor**	***N***	**All**	**Discussion** **no**	**Discussion** **yes**	**Univariable OR (95 % CI)**	***p*-value**	Multivariable OR (95 % CI)*	***p*-value**
***N***	474	474	205	269				

***Patients’ factors***								
**Advanced age (e.g. >80 years) yes, *n* (%)**	422	408 (96.7 %)	171 (95.0 %)	237 (97.9 %)	2.49 (0.82, 7.58)	0.107	2.9 (0.92, 9.1)	0.069
**Young age (e.g. <50 years) yes, *n* (%)**	417	33 (7.9 %)	9 (5.1 %)	24 (10.0 %)	2.1 (0.95, 4.63)	0.067	2.34 (1.05, 5.25)	0.038
**Elective planned admission yes, *n* (%)**	418	64 (15.3 %)	23 (13.0 %)	41 (17.0 %)	1.37 (0.79, 2.38)	0.261	1.51 (0.85, 2.65)	0.157
**Inpatient status yes, *n* (%)**	420	134 (31.9 %)	49 (27.4 %)	85 (35.3 %)	1.45 (0.95, 2.2)	0.087	1.61 (1.04, 2.5)	0.034
**High expected perioperative complication risk, yes, *n* (%)**	419	386 (92.1 %)	155 (88.1 %)	231 (95.1 %)	2.61 (1.25, 5.45)	0.011	3.14 (1.46, 6.78)	0.004
**Low expected perioperative complication risk yes, *n* (%)**	422	39 (9.2 %)	13 (7.3 %)	26 (10.7 %)	1.53 (0.76, 3.07)	0.231	1.76 (0.86, 3.58)	0.12
**Pre-existing complete need for care (e.g. nursing home) yes, *n* (%)**	420	363 (86.4 %)	142 (79.3 %)	221 (91.7 %)	2.88 (1.61, 5.16)	<0.001	3 (1.65, 5.48)	<0.001
**Low expected quality of life after surgery yes, *n* (%)**	419	379 (90.5 %)	151 (84.8 %)	228 (94.6 %)	3.14 (1.57, 6.27)	0.001	3.78 (1.84, 7.75)	<0.001
**Significant comorbidities (e.g. advanced cardiac or pulmonary disease) yes, *n* (%)**	421	388 (92.2 %)	152 (85.4 %)	236 (97.1 %)	5.77 (2.44, 13.62)	<0.001	6.1 (2.53, 14.73)	<0.001
**High iatrogenic risk of circulatory arrest (heart surgery, intrathoracic procedures) yes, *n* (%)**	420	401 (95.5 %)	163 (91.1 %)	238 (98.8 %)	7.79 (2.23, 27.16)	0.001	6.93 (1.97, 24.44)	0.003
**Cognitive impairment or dementia yes, *n* (%)**	418	327 (78.2 %)	120 (68.2 %)	207 (85.5 %)	2.76 (1.71, 4.45)	<0.001	3.04 (1.84, 5.02)	<0.001
**Presence of a documented advance directive yes, *n* (%)**	419	330 (78.8 %)	126 (71.6 %)	204 (84.0 %)	2.08 (1.29, 3.33)	0.003	2.04 (1.25, 3.31)	0.004
**Known malignancy or hematologic cancer, *n* (%)**	421	294 (69.8 %)	105 (58.7 %)	189 (78.1 %)	2.51 (1.64, 3.85)	<0.001	2.49 (1.6, 3.85)	<0.001

***Anaesthetists’ preferences***								
**After surgery, the patient's resuscitation status should be changed to the original resuscitation status yes, *n* (%)**	386	326 (84.5 %)	139 (86.9 %)	187 (82.7 %)	0.72 (0.41, 1.29)	0.271	0.75 (0.42, 1.35)	0.332
**In the case of perioperative resuscitation with subsequent ROSC, postoperative intensive care measures should be continued more intensively/aggressively, as the complication occurred in a surgical context yes, *n* (%)**	387	144 (37.2 %)	60 (37.3 %)	84 (37.2 %)	1 (0.66, 1.51)	0.984	0.92 (0.6, 1.41)	0.691
**The decision to discontinue therapy after perioperative resuscitation should involve both the surgical and anaesthesiology teams, with equal weighting of opinion yes, *n* (%)**	388	347 (89.4 %)	145 (90.1 %)	202 (89.0 %)	0.89 (0.46, 1.73)	0.734	0.75 (0.37, 1.49)	0.409
**When determining the perioperative resuscitation status, there are often interdisciplinary conflicts between surgery and anaesthesia yes, *n* (%)**	390	225 (57.7 %)	103 (63.6 %)	122 (53.5 %)	0.66 (0.44, 1)	0.048	0.73 (0.48, 1.11)	0.144
**An interdisciplinary determination/discussion of the resuscitation status between anaesthesia and surgery should be standard practice yes, *n* (%)**	387	351 (90.7 %)	137 (85.6 %)	214 (94.3 %)	2.76 (1.35, 5.64)	0.005	2.78 (1.35, 5.76)	0.006
**In view of the ageing population, perioperative resuscitation status should be discussed more frequently yes, *n* (%)**	387	375 (96.9 %)	153 (95.0 %)	222 (98.2 %)	2.9 (0.86, 9.81)	0.086	3.48 (1, 12.06)	0.049

Data presented as *n* (%) or mean (95% CI) unless otherwise specified. Abbreviations: **OR,** Odds Ratio; **SD**, standard deviation; **n**, number; **ROSC,** return of spontaneous circulation.

* Adjusted for age, gender.

### Primary endpoint: Preoperative code status discussion in medium-risk patients (ASA 3–4)

Regarding the primary endpoint, 269 of 474 respondents (56.8 %) reported routinely discussing code status preoperatively with patients in the medium-risk group (ASA 3–4) (Table 2). Participants who reported engaging in these discussions were significantly older (mean [±SD] age 47.8 [±10.7] vs. 44.6 [11.3], adjusted OR 1.03 [95 % CI 1.01–1.05], *p* < 0.001), more often female (130/269 [48.3 %] vs. 72/205 [35.1 %], adjusted OR 1.94 [95 % CI 1.32–2.84], *p* = 0.001), more frequently board-certified (232/269 [86.2 %] vs. 160/205 [78.0 %], adjusted OR 1.98 [95 % CI 1.21–3.24], *p* = 0.006) and had more professional experience (mean [±SD] 20.7 [±10.6] vs. 17.4 [10.8] years, adjusted OR 1.03 [95 % CI 1.02–1.05], *p* = 0.001) compared to those who did not (Table 1a).

**Table 2:** Primary and secondary outcomes.

**Primary endpoint: frequency of preoperative code status discussion for patients ASA 3–4**	**Sample Size**	***n* (%)**
**Frequency of code status discussion, ASA 3–4, *n* (%)**	474	269 (56.8 %)

**Secondary endpoints**	**Sample Size**	***n* (%)**

***Frequency of preoperative code status discussion in low and high-risk patients***		
**Frequency of code status discussion, ASA 1–2, *n* (%)**	474	21 (4.4 %)
**Frequency of code status discussion, ASA 5, *n* (%)**		402 (84.8 %)
***Frequency of preoperative discussion of ICU admission and severe complications***		
**Frequency of code status discussion, ASA 1–2, *n* (%)**	467	63 (13.5 %)
**Frequency of code status discussion, ASA 3–4, *n* (%)**		363 (77.7 %)
**Frequency of code status discussion, ASA 5, *n* (%)**		420 (89.9 %)
***Frequency of preoperative discussion of postoperative therapy limitations***		
**Frequency of code status discussion, ASA 1–2, *n* (%)**	461	14 (3.0 %)
**Frequency of code status discussion, ASA 3–4, *n* (%)**		218 (47.3 %)
**Frequency of code status discussion, ASA 5, *n* (%)**		372 (80.7 %)
***Frequency of preoperative code status discussion in patients with DNR orders***		
**Preoperative code status discussion in patients with DNR orders, yes, *n* (%)**	417	318 (76.3 %)

Abbreviations: ***n***, number, **DNR** Do Not Resuscitate order, **ICU** Intensive-care Unit, **ASA** American Society of Anesthesiologists classification.

These participants also reported greater CPR experience, with 169/269 (62.8 %) having performed more than 50 resuscitations compared to 93/205 (45.4 %) in the non-discussion group (adjusted OR 2.99 [95 % CI 1.39–6.42], *p* = 0.005). In addition, physicians who reported to routinely assess advance directives before interventions were significantly more likely to discuss code status (174/242 [71.9 %] vs. 60/180 [33.3 %], adjusted OR 5.33 [95 % CI 3.46–8.21], *p* < 0.001). They were also more likely to be aware of institutional teaching on code status (48/243 [19.8 %] vs. 13/180 [7.2 %], adjusted OR 2.93 [95 % CI 1.51–5.68], *p* = 0.001), although overall, only 14.4 % of all respondents reported that such teaching was available at their institution. Conversely, participants were less likely to discuss code status when they were concerned about frightening patients (113/180 [62.8 %] vs. 116/243 [47.7 %], adjusted OR 0.53 [95 % CI 0.35–0.79], *p* = 0.002).

Regarding responsibility, 381/451 (84.5 %) of participants believed that both anaesthetists and surgeons should share responsibility for code status discussions. No significant difference was found between groups in the estimation of cardiac arrest survival (Table 1a).

Among patient-related factors, the five scenarios most frequently cited by participants as reasons to initiate code status discussions were: advanced patient age (96.7 %), high iatrogenic risk for POCA (95.5 %), significant comorbidities (92.2 %), high risk of perioperative complications (92.1 %), and low anticipated postoperative quality of life (90.5 %). Other significant differences between the ‘discussion yes’ and ‘discussion no’ groups were observed for several factors, including preexisting high-level care dependence, cognitive impairment, known malignancy, presence of advance directives, high iatrogenic risk of POCA, and low expected postoperative quality of life (Table 1b).

Regarding the anaesthetists’ attitudes, 375/387 participants (96.9 %) agreed that code status should be addressed more frequently in an aging society, and 351/387 (90.7 %) supported interdisciplinary code status discussions as standard of care. Participants in the discussion group were significantly more likely to support interdisciplinary discussions (214/227 [94.3 %] vs. 137/160 [85.6 %], adjusted OR 2.78 [95 % CI 1.35–5.76], *p* = 0.006) (Table 1b).

### Secondary endpoint: Preoperative discussion of severe complications and ICU admission

Secondary outcomes by ASA risk group are shown in Table 2. Overall, 363/467 (77.7 %) of participants reported routinely discussing severe complications and ICU admissions with ASA 3–4 patients. These discussions were significantly more common among participants with greater professional experience, those who routinely assessed advance directives and those aware of institutional education on this topic. Additional significant group differences were observed in CPR experience (both overall and perioperative) and concerns about frightening patients during code status discussions (Supplementary Tables 2 and 3).

### Secondary endpoint: Preoperative discussion of therapy limitations

Regarding the preoperative discussion of therapy limitations, 3 % of participants reported routinely addressing this topic with ASA 1–2 patients, 47.3 % with ASA 3–4 patients, and 80.7 % with ASA 5 patients (Table 2).

### Secondary endpoint: Preoperative discussion of code status in patients with DNR orders

Among 417 participants, 318 (76.3 %) reported discussing code status with patients who have a preexisting DNR order. This discussion was more likely when physicians routinely assessed advance directives (194/318 [61.0 %] vs. 36/99 [36.4 %], adjusted OR 2.72 [95 % CI 1.7–4.34], *p* < 0.001) or when they were aware of institutional teaching on the topic (55/318 [17.3 %] vs. 6/99 [6.1 %], adjusted OR 3.04 [95 % CI 1.26–7.36], *p* = 0.013). As with the primary endpoint, physicians were less likely to discuss code status when being concerned about frightening their patients (64/99 [64.6 %] vs. 162/318 [50.9 %], adjusted OR 0.58 [95 % CI 0.36–0.93], *p* = 0.024) (Supplementary Table 1). Regarding patient-related factors and anaesthetists’ beliefs, 70.4 % of participants agreed that a DNR order should be respected during the perioperative period. However, 32.3 % found such orders illogical in this context, 21.7 % believed they should not be respected and 21.3 % felt they should play no role perioperatively (Supplementary Table 1).

## Discussion

In this national web-based survey of 474 anaesthetists, 56.8 % reported routinely conducting preoperative code status discussions with patients at medium risk (ASA 3–4). Key factors leading to code status discussions included older age, more years of professional experience, greater exposure to CPR, routine assessment of advance directives, awareness of institutional teaching on the topic, and less concern that such discussions would distress patients. For the key secondary endpoint, 76.3 % of respondents reported routinely discussing code status with patients who had existing DNR orders.

Previous research suggests that active code status discussion or documentation in the perioperative setting remains insufficient in clinical practice.^18^, ^29^ A systematic review highlighted that communication regarding postoperative care and potential complications is generally rare.^30^ In contrast, our findings indicate that anaesthetists frequently engage in such discussions with high-risk (ASA 5) and medium-risk (ASA 3–4) patients, though far less often with low-risk patients (ASA 1–2). To the best of our knowledge, this represents the first comprehensive survey exploring these practices specifically among anaesthetists.

Several valuable insights emerged. First, experienced physicians were more likely to report routinely conducting code status discussions, aligning with prior findings that senior physicians tend to be more aware of potential ethical issues when performing CPR in a futile situation. Previous research found that attending physicians demonstrated greater knowledge about end-of-life than residents,^31^ and that residents felt less confident conducting such discussions.^32^ This suggests that senior physicians’ broader exposure to complex clinical and ethical situations may enhance their ability to navigate sensitive communication, while residents may benefit from guidance and role modelling by more experienced senior physicians when conducting code status discussions.

Second, physicians with greater CPR experience were more likely to report routinely conducting preoperative code status discussions. This may reflect greater familiarity with actual clinical resuscitation outcomes, as previous research has shown that clinicians with greater CPR experience are more likely to forgo potentially futile resuscitation interventions.^19^ These findings underscore the need for physicians to accurately understand CPR outcomes to ensure patient-centred code status discussions.

Third, our results suggest that physicians who were afraid of frightening their patients by discussing code status were more likely to avoid this topic. This reflects known barriers such as discomfort with discussing mortality, fear of harming the doctor-patient relationship.^33^ However, a randomized controlled multicentre trial found that such discussions did not increase patient anxiety when conducted using a shared decision-making approach^34^ – though this has not yet been studied in the perioperative setting to date.

Fourth, only 14.4 % of participants reported receiving formal resident training on perioperative code status discussions, with higher awareness among those who routinely conduct them. This is consistent with previous studies showing that perioperative providers often receive insufficient training in how to handle DNR orders.^35^, ^36^, ^37^ Structured training has been shown to improve residents’ performance in these discussions,^37^, ^38^, ^39^ supporting the need for such education during residency.

Fifth, most physicians (76.3 %) reported discussing code status with patients when confronted with DNR orders, and 70 % stated they would respect a DNR order in the perioperative setting. Compared with earlier studies, fewer anaesthetists viewed perioperative DNR orders as illogical or automatically suspended,^20^, ^21^ possibly reflecting greater awareness of ASA guidelines.^16^, ^40^ Establishing a clear approach and shared team attitude toward managing DNR orders is essential for consistency in the perioperative setting.

Lastly, literature supports a structured approach to managing surgical patients with DNR orders. This includes avoiding automatic reversal of DNR status, encouraging open exchange between anaesthetists and surgeons, defining clear perioperative goals of care, and thoroughly documenting any temporary modifications, as well as the patient’s overall goals of care.^41^ Existing DNR orders require reconsideration rather than automatic suspension, ideally through early joint discussions between anaesthetists and surgeons during surgical planning.^42^

### Strengths and limitations

This survey’s strength lies in its broad inclusion of both teaching and non-teaching hospitals across Switzerland and its high completion rate (95.6 %), providing a representative overview of current national practices. Limitations include an unclear response rate, as the total number of individuals who received or viewed the invitation could not be determined due to its design as an open, web-based survey distributed via institutional networks without a defined recipient list. A further limitation is the underrepresentation of residents, which may limit generalizability. Moreover, these findings may not be directly transferable to other healthcare systems due to variations in clinical practice, healthcare policies, and cultural norms. Finally, as an observational survey, the study cannot establish causality. We did not apply a Bonferroni correction, as our primary goal was to explore potential associations rather than to control for multiple testing in a confirmatory setting. Consequently, this analysis should be considered exploratory and hypothesis-generating rather than definitive. As with all self-reported survey data, recall and response bias cannot be excluded. Reported practices may not fully correspond to actual clinical behaviour, and social desirability may have influenced responses. Future studies using observational or mixed-method approaches are warranted to validate these findings.

## Conclusions

This nationwide survey highlights current practices among Swiss anaesthetists regarding perioperative code status discussions and DNR management. While over half reported routinely discussing code status with medium risk (ASA 3–4) patients, such discussions were more frequent in high-risk cases (ASA 5) and less common than in low-risk cases (ASA 1–2). Physicians who actively engaged in these conversations reported to be more experienced, were more likely to be female, regularly assessed advance directives during premedication visits, and stated to be less concerned about causing distress to patients. Although most respondents supported respecting DNR orders perioperatively, only a minority reported institutional teaching on the topic. These findings underscore the need for clearer guidelines, interdisciplinary collaboration, and targeted training to support patient-centred, consistent code status discussions in perioperative care.

## Authors’ contributions

SZ, FG and SH were the main contributors regarding conceptualization, methodology, acquisition, analysis and interpretation of the data, as well as writing, editing and visualizing the manuscript. SH was the supervisor and administrator of the project and provided funding. SA, BB, AA, KT, RS, TG and LS made substantial contributions to the data acquisition and to the editing and revision of the manuscript. AR, ML, SG and CB provided resources for the data acquisition and revised the manuscript substantially. All authors read and approved the final manuscript.

## CRediT authorship contribution statement

**Samuel K. Zumbrunn:** Writing – original draft, Visualization, Investigation, Formal analysis, Data curation, Conceptualization. **Flavio Gössi:** Writing – original draft, Visualization, Investigation, Formal analysis, Data curation, Conceptualization. **Benjamin Bissmann:** Writing – review & editing, Data curation, Conceptualization. **Armon Arpagaus:** Writing – review & editing, Data curation, Conceptualization. **Sebastian Gross:** Writing – review & editing, Data curation, Conceptualization. **Christoph Becker:** Writing – review & editing, Data curation, Conceptualization. **Simon A. Amacher:** Writing – review & editing, Data curation, Conceptualization. **Raoul Sutter:** Writing – review & editing, Data curation, Conceptualization. **Kai Tisljar:** Writing – review & editing, Data curation, Conceptualization. **Ariane Rossi:** Writing – review & editing, Data curation, Conceptualization. **Marc Lüthy:** Writing – review & editing, Data curation, Conceptualization. **Luzius A. Steiner:** Writing – review & editing, Data curation, Conceptualization. **Thierry Girard:** Writing – review & editing, Data curation, Conceptualization. **Sabina Hunziker:** Writing – review & editing, Writing – original draft, Supervision, Resources, Project administration, Methodology, Funding acquisition, Formal analysis, Data curation, Conceptualization.

## Declaration of competing interest

Prof. Sabina Hunziker and her research team are supported by the Swiss National Foundation (SNF) (Ref 10001C_192850/1) and the Swiss Society of General Internal Medicine (SSGIM), and the Gottfried Julia Bangerter-Rhyner Foundation (8472/HEG-DSV) and the Swiss Society of General Internal Medicine (SSGIM). Armon Arpagaus was supported by the Gottfried und Julia Bangerter-Rhyner-Stiftung (YCTR 6/23).
