# Supplementary material for: Challenges in perioperative code status management: a national survey among Swiss anaesthetists
Source: Resusc Plus. 2025 Nov 22;27:101174. doi: 10.1016/j.resplu.2025.101174 (PMC12722988; doi:10.1016/j.resplu.2025.101174)
Supplement: Supplementary Data 1 [file mmc1.docx]

**Appendix A: Supplementary Data**

**Challenges in Perioperative Code Status Management: A National Survey Among Swiss Anaesthetists**

Samuel K. Zumbrunn, MD^1*^; Flavio Gössi, MD^1,4*^, Benjamin Bissmann, MD^1^; Armon Arpagaus, MD^1,4^; Sebastian Gross, Ph.D^1^; Christoph Becker, MD^1,2,4^; Simon A. Amacher, MD^5, 6^, Ariane Rossi, MD^7^, Marc Lüthy, MD^7, 8^, Luzius A. Steiner, MD Ph.D^3, 7^, Thierry Girard, MD^3, 7^ and Sabina Hunziker, MD MPH^1,3, 9^

^1^ Medical Communication and Psychosomatic Medicine, University Hospital Basel, Basel, Switzerland

^2^ Medical Outpatient Department, University Hospital Basel, Basel, Switzerland

^3^ Faculty of Medicine, University of Basel, Basel, Switzerland

^4^ Division of Internal Medicine, University Hospital Basel, Basel, Switzerland

^5^ Department of Anaesthesiology and Intensive Care Medicine, Medical Center-University of Freiburg, Faculty of Medicine, University of Freiburg, Freiburg im Breisgau, Germany

^6^ Intensive Care Unit, Department of Acute Medicine, University Hospital Basel, Basel, Switzerland

^7^ Clinic for Anaesthesia, Intermediate Cre, Prehosptial Emergency Medicine and Pain Therapy, University Hospital Basel and University of Basel, Basel, Switzerland

^8^ Sanität, Rettung Basel-Stadt, Justiz- und Sicherheitsdepartement des Kantons Basel-Stadt, Basel, Switzerland

^9^ Post-Intensive Care Clinic, University Hospital Basel, Basel, Switzerland

**These authors are equally contributing to this work*

**Supplement 1. Anaesthetists conducting perioperative CSD in case of documented DNR.**

| Factor | Categories | N | All | Discussion no | Discussion yes | Univariable OR (95% CI) | p-value | Multivariable OR (95% CI)* | p-value |
| --- | --- | --- | --- | --- | --- | --- | --- | --- | --- |
| N |  |  | 417 | 99 | 318 |  |  |  |  |
| *Demographic factors of clinician* | | | | | | | | | |
| Age (years), mean (SD) |  | 417 | 46.1 (11.1) | 44.6 (10.7) | 46.6 (11.2) | 1.02 (1, 1.04) | 0.131 | 1.02 (0.99, 1.04) | 0.143 |
| Gender, n (%) | Female | 417 | 178 (42.7%) | 44 (44.4%) | 134 (42.1%) | 0.91 (0.58, 1.43) | 0.685 | 0.96 (0.61, 1.52) | 0.864 |
| Hospital size, n (%) | Centre hospital with maximum care (e.g. university hospital), | 417 | 171 (41.0%) | 38 (38.4%) | 133 (41.8%) | (Ref.) |  | (Ref.) |  |
|  | cantonal hospital |  | 109 (26.1%) | 26 (26.3%) | 83 (26.1%) | 0.91 (0.52, 1.61) | 0.751 | 0.83 (0.46, 1.49) | 0.534 |
|  | Regional hospital |  | 79 (18.9%) | 22 (22.2%) | 57 (17.9%) | 0.74 (0.4, 1.36) | 0.334 | 0.59 (0.31, 1.15) | 0.12 |
|  | Surgeon in private practice |  | 58 (13.9%) | 13 (13.1%) | 45 (14.2%) | 0.99 (0.48, 2.02) | 0.976 | 0.72 (0.32, 1.58) | 0.405 |
| Function, n (%) | Resident | 417 | 86 (20.6%) | 20 (20.2%) | 66 (20.8%) | (Ref.) |  | (Ref.) |  |
|  | Attending physician |  | 131 (31.4%) | 35 (35.4%) | 96 (30.2%) | 0.83 (0.44, 1.56) | 0.567 | 0.83 (0.44, 1.57)** | 0.573 |
|  | Consultant |  | 126 (30.2%) | 29 (29.3%) | 97 (30.5%) | 1.01 (0.53, 1.94) | 0.968 | 1.01 (0.53, 1.94)** | 0.98 |
|  | Head physician |  | 74 (17.7%) | 15 (15.2%) | 59 (18.6%) | 1.19 (0.56, 2.54) | 0.649 | 1.18 (0.55, 2.53)** | 0.672 |
| Speciality title, yes, n (%) |  | 417 | 345 (82.7%) | 80 (80.8%) | 265 (83.3%) | 1.19 (0.66, 2.12) | 0.562 | 1.17 (0.65, 2.11)** | 0.596 |
| Clinical experience in years, mean, SD |  | 417 | 19.1 (10.7) | 18.0 (10.3) | 19.4 (10.9) | 1.01 (0.99, 1.03) | 0.246 | 1.01 (0.99, 1.03)** | 0.264 |
| *Physician related factors* | | | | | | | | | |
| Premedication consultations, mean, SD |  | 417 | 11.1 (8.6) | 12.3 (10.2) | 10.7 (8.1) | 0.98 (0.95, 1) | 0.094 | 0.98 (0.95, 1) | 0.072 |
| Anaesthesia procedures per week, mean, SD |  | 417 | 19.9 (14.2) | 20.4 (16.2) | 19.7 (13.5) | 1 (0.98, 1.01) | 0.652 | 1 (0.98, 1.01) | 0.558 |
| Number of performed perioperative CPR, n (%) | Below 10 | 417 | 253 (60.7%) | 60 (60.6%) | 193 (60.7%) | (Ref.) |  | (Ref.) |  |
|  | 10 - 50 |  | 144 (34.5%) | 36 (36.4%) | 108 (34.0%) | 0.93 (0.58, 1.5) | 0.774 | 0.79 (0.47, 1.33) | 0.368 |
|  | Over 50 |  | 20 (4.8%) | 3 (3.0%) | 17 (5.3%) | 1.76 (0.5, 6.22) | 0.379 | 1.39 (0.38, 5.07) | 0.622 |
| Number of performed CPR in general, n (%) | Below 10 | 417 | 31 (7.4%) | 9 (9.1%) | 22 (6.9%) | (Ref.) |  | (Ref.) |  |
|  | 10 - 50 |  | 156 (37.4%) | 41 (41.4%) | 115 (36.2%) | 1.15 (0.49, 2.69) | 0.752 | 1.03 (0.43, 2.47) | 0.953 |
|  | Over 50 |  | 230 (55.2%) | 49 (49.5%) | 181 (56.9%) | 1.51 (0.65, 3.49) | 0.334 | 1.26 (0.51, 3.1) | 0.614 |
| Estimated survival rate after perioperative CPR, mean (SD) |  | 417 | 40.1 (22.4) | 41.3 (21.8) | 39.7 (22.6) | 1 (0.99, 1.01) | 0.536 | 1 (0.99, 1.01) | 0.546 |
| *Factors related to the perioperative discussion* | | | | | | | | | |
| Surgeon responsible for code status discussions yes, n (%) | Anaesthetist | 417 | 49 (11.8%) | 6 (6.1%) | 43 (13.5%) | (Ref.) |  | (Ref.) |  |
|  | Both Anaesthetist and Surgeon |  | 353 (84.7%) | 86 (86.9%) | 267 (84.0%) | 0.43 (0.18, 1.05) | 0.065 | 0.45 (0.18, 1.09) | 0.077 |
|  | Surgeon |  | 15 (3.6%) | 7 (7.1%) | 8 (2.5%) | 0.16 (0.04, 0.6) | 0.007 | 0.17 (0.05, 0.66) | 0.01 |
| Advanced directives (AD) assessed prior to intervention, n (%) |  | 417 | 230 (55.2%) | 36 (36.4%) | 194 (61.0%) | 2.74 (1.72, 4.37) | <0.001 | 2.72 (1.7, 4.34) | <0.001 |
| Are prior AD relevant in a perioperative phase, yes, n (%) |  | 417 | 295 (70.7%) | 62 (62.6%) | 233 (73.3%) | 1.64 (1.02, 2.64) | 0.043 | 1.59 (0.98, 2.56) | 0.059 |
| Decisional preference in perioperative CSD, n, (%) | Physician | 415 | 23 (5.5%) | 9 (9.1%) | 14 (4.4%) | (Ref.) |  | (Ref.) |  |
|  | Physician/patient |  | 323 (77.8%) | 74 (74.7%) | 249 (78.8%) | 2.16 (0.9, 5.2) | 0.085 | 2.19 (0.91, 5.28) | 0.081 |
|  | Patient |  | 69 (16.6%) | 16 (16.2%) | 53 (16.8%) | 2.13 (0.78, 5.83) | 0.141 | 2.06 (0.74, 5.69) | 0.165 |
| Teaching for residents, yes, n (%) |  | 417 | 61 (14.6%) | 6 (6.1%) | 55 (17.3%) | 3.24 (1.35, 7.78) | 0.008 | 3.04 (1.26, 7.36) | 0.013 |
| Do you think perioperative CSD scare the patient, yes, n (%) |  | 417 | 226 (54.2%) | 64 (64.6%) | 162 (50.9%) | 0.57 (0.36, 0.91) | 0.018 | 0.58 (0.36, 0.93) | 0.024 |
| *Anaesthetists’ views on CSD in DNR patients* | | | | | | | | | |
| Estimated frequency of patients with a preexisting DNR order n, (%) |  |  |  |  |  |  |  |  |  |
| Less than 25% |  | 417 | 355 (85.1%) | 78 (78.8%) | 277 (87.1%) | (Ref.) |  | (Ref.) |  |
| 25-50% |  |  | 59 (14.1%) | 20 (20.2%) | 39 (12.3%) | 0.55 (0.3, 1) | 0.048 | 0.58 (0.32, 1.07) | 0.082 |
| 50-75% |  |  | 2 (0.5%) | 0 (0.0%) | 2 (0.6%) | NA |  | NA |  |
| Over 75% |  |  | 1 (0.2%) | 1 (1.0%) | 0 (0.0%) | NA |  | NA |  |
| A DNR status is illogical perioperatively, as anaesthesia naturally involves ‘resuscitation measures’ such as intubation and pressure support yes, n (%) |  | 405 | 131 (32.3%) | 34 (35.1%) | 97 (31.5%) | 0.85 (0.53, 1.38) | 0.514 | 0.85 (0.53, 1.38) | 0.514 |
| Perioperatively, anaesthesia and surgery interfere with the cardiopulmonary system; therefore, the surgical team must be able to use all necessary skills regardless of a patient's pre-existing resuscitation status yes, n (%) |  | 402 | 194 (48.3%) | 47 (49.0%) | 147 (48.0%) | 0.96 (0.61, 1.52) | 0.875 | 0.93 (0.58, 1.49) | 0.766 |
| A DNR status must be respected perioperatively, as decisions on resuscitation are based on the patient's values and not solely on the judgment of the surgical team yes, n (%) |  | 405 | 285 (70.4%) | 65 (67.0%) | 220 (71.4%) | 1.23 (0.75, 2.01) | 0.406 | 1.2 (0.74, 1.97) | 0.458 |
| A DNR status should not be respected perioperatively, as the probability of successful resuscitation is higher in a monitored operating room yes, n (%) |  | 401 | 87 (21.7%) | 23 (24.2%) | 64 (20.9%) | 0.83 (0.48, 1.43) | 0.496 | 0.81 (0.47, 1.4) | 0.453 |
| A DNR status plays no role perioperatively, as it is difficult to differentiate between spontaneous and anaesthesia-induced cardiorespiratory arrest yes, n (%) |  | 403 | 86 (21.3%) | 19 (19.8%) | 67 (21.8%) | 1.13 (0.64, 2) | 0.672 | 1.09 (0.62, 1.94) | 0.762 |
| Patients who are able to consent to a surgical and anaesthesiologic procedure, are as well able to consent or to refuse a perioperative resuscitation yes, n (%) |  | 403 | 343 (85.1%) | 80 (82.5%) | 263 (85.9%) | 1.3 (0.7, 2.4) | 0.403 | 1.35 (0.73, 2.52) | 0.34 |

**Supplementary Table 1: Secondary outcome: Anaesthetists conducting perioperative CSD in case of documented DNR**

Data presented as n (%) or mean (SD) unless otherwise specified. *adjusted for age, gender, **adjusted for gender only due to collinearity with age; Abbreviations: **OR,** Odds Ratio; **SD**, standard deviation; **n**, number; **CPR** cardio-pulmonary resuscitation; **AD** advance directive; **CSD** Code Status Discussion; **DNR** Do Not Resuscitate order.

**Supplement 2. Anaesthetists conducting perioperative discussions about intensive care admissions and severe complications**

| Factor | Categories | N | All | Discussion no | Discussion yes | Univariable OR (95% CI) | p-value | Multivariable OR (95% CI)* | p-value |
| --- | --- | --- | --- | --- | --- | --- | --- | --- | --- |
| *Demographic factors of clinicians* | | | | | | | | | |
| N |  | 467 | 467 | 104 | 363 |  |  |  |  |
| Age (years), mean (SD) |  | 467 | 46.5 (11.0) | 44.7 (10.9) | 47.0 (11.0) | 1.02 (1, 1.04) | 0.059 | 1.02 (1, 1.04) | 0.046 |
| Gender, n (%) | Female | 467 | 198 (42.4%) | 41 (39.4%) | 157 (43.3%) | 1.17 (0.75, 1.83) | 0.486 | 1.25 (0.79, 1.96) | 0.338 |
| Hospital size, n (%) | Centre hospital with maximum care (e.g. university hospital), | 467 | 189 (40.5%) | 44 (42.3%) | 145 (39.9%) | (Ref.) |  | (Ref.) |  |
|  | cantonal hospital |  | 122 (26.1%) | 26 (25.0%) | 96 (26.4%) | 1.12 (0.65, 1.94) | 0.685 | 1 (0.57, 1.74) | 0.986 |
|  | Regional hospital |  | 90 (19.3%) | 17 (16.3%) | 73 (20.1%) | 1.3 (0.7, 2.44) | 0.408 | 1 (0.51, 1.95) | 0.998 |
|  | Surgeon in private practice |  | 66 (14.1%) | 17 (16.3%) | 49 (13.5%) | 0.87 (0.46, 1.67) | 0.685 | 0.62 (0.3, 1.27) | 0.192 |
| Function, n (%) | Resident | 467 | 93 (19.9%) | 28 (26.9%) | 65 (17.9%) | (Ref.) |  | (Ref.) |  |
|  | Attending physician |  | 142 (30.4%) | 34 (32.7%) | 108 (29.8%) | 1.37 (0.76, 2.46) | 0.295 | 1.35 (0.75, 2.43)** | 0.317 |
|  | Consultant |  | 153 (32.8%) | 30 (28.8%) | 123 (33.9%) | 1.77 (0.97, 3.21) | 0.062 | 1.82 (1, 3.31)** | 0.051 |
|  | Head physician |  | 79 (16.9%) | 12 (11.5%) | 67 (18.5%) | 2.41 (1.13, 5.13) | 0.023 | 2.58 (1.2, 5.56)** | 0.016 |
| Specialty title, yes, n (%) |  | 467 | 389 (83.3%) | 83 (79.8%) | 306 (84.3%) | 1.36 (0.78, 2.37) | 0.28 | 1.4 (0.8, 2.46)** | 0.237 |
| Clinical experience in years, mean, SD |  | 467 | 19.4 (10.7) | 17.5 (10.6) | 19.9 (10.7) | 1.02 (1, 1.04) | 0.047 | 1.02 (1, 1.04)** | 0.038 |
| Physician related factors | | | | | | | | | |
| Premedication consultations, mean, SD |  | 467 | 11.3 (8.9) | 10.8 (9.0) | 11.5 (8.9) | 1.01 (0.98, 1.04) | 0.475 | 1.01 (0.98, 1.03) | 0.543 |
| Anaesthesia procedures per week, mean, SD |  | 467 | 20.2 (14.0) | 19.5 (16.2) | 20.4 (13.3) | 1 (0.99, 1.02) | 0.585 | 1 (0.99, 1.02) | 0.645 |
| Number of performed perioperative CPR, n (%) | Below 10 | 467 | 275 (58.9%) | 71 (68.3%) | 204 (56.2%) | (Ref.) |  | (Ref.) |  |
|  | 10 - 50 |  | 169 (36.2%) | 32 (30.8%) | 137 (37.7%) | 1.49 (0.93, 2.38) | 0.096 | 1.41 (0.84, 2.35) | 0.19 |
|  | Over 50 |  | 23 (4.9%) | 1 (1.0%) | 22 (6.1%) | 7.66 (1.01, 57.84) | 0.048 | 6.93 (0.9, 53.33) | 0.063 |
| Number of performed CPR in general, n (%) | Below 10 | 467 | 36 (7.7%) | 12 (11.5%) | 24 (6.6%) | (Ref.) |  | (Ref.) |  |
|  | 10 - 50 |  | 169 (36.2%) | 48 (46.2%) | 121 (33.3%) | 1.26 (0.58, 2.72) | 0.555 | 1.21 (0.55, 2.68) | 0.634 |
|  | Over 50 |  | 262 (56.1%) | 44 (42.3%) | 218 (60.1%) | 2.48 (1.15, 5.32) | 0.02 | 2.41 (1.05, 5.53) | 0.038 |
| Estimated survival rate after perioperative CPR, mean (SD) |  | 420 | 40.0 (22.3) | 38.5 (20.9) | 40.5 (22.7) | 1 (0.99, 1.01) | 0.446 | 1 (0.99, 1.01) | 0.458 |
| *Factors related to the perioperative discussion* | | | | | | | | | |
| Surgeon responsible for code status discussions yes, n (%) | Anaesthetist | 451 | 53 (11.8%) | 14 (14.4%) | 39 (11.0%) | (Ref.) |  | (Ref.) |  |
|  | Both Anaesthetist and Surgeon |  | 381 (84.5%) | 76 (78.4%) | 305 (86.2%) | 1.44 (0.74, 2.79) | 0.279 | 1.46 (0.75, 2.86) | 0.268 |
|  | Surgeon |  | 17 (3.8%) | 7 (7.2%) | 10 (2.8%) | 0.51 (0.16, 1.61) | 0.252 | 0.55 (0.17, 1.75) | 0.313 |
| Advance directives (AD) assessed prior to intervention, n (%) |  | 422 | 234 (55.5%) | 33 (35.1%) | 201 (61.3%) | 2.93 (1.81, 4.72) | <0.001 | 2.88 (1.78, 4.65) | <0.001 |
| Are prior AD relevant in a perioperative phase, yes, n (%) |  | 423 | 299 (70.7%) | 58 (61.7%) | 241 (73.3%) | 1.7 (1.05, 2.75) | 0.031 | 1.64 (1.01, 2.67) | 0.046 |
| Decisional preference in perioperative CSD, n, (%) | Physician | 448 | 28 (6.2%) | 8 (8.2%) | 20 (5.7%) | (Ref.) |  | (Ref.) |  |
|  | Physician/patient |  | 346 (77.2%) | 74 (76.3%) | 272 (77.5%) | 1.47 (0.62, 3.47) | 0.379 | 1.51 (0.64, 3.59) | 0.349 |
|  | Patient |  | 74 (16.5%) | 15 (15.5%) | 59 (16.8%) | 1.57 (0.58, 4.26) | 0.373 | 1.49 (0.55, 4.09) | 0.434 |
| Teaching for residents, yes, n (%) |  | 423 | 61 (14.4%) | 5 (5.3%) | 56 (17.0%) | 3.65 (1.42, 9.4) | 0.007 | 3.36 (1.3, 8.73) | 0.013 |
| Do you think perioperative CSD scare the patient, yes, n (%) |  | 423 | 229 (54.1%) | 68 (72.3%) | 161 (48.9%) | 0.37 (0.22, 0.6) | <0.001 | 0.37 (0.22, 0.62) | <0.001 |

**Supplementary Table 2.** Secondary Endpoint: Anaesthetists conducting perioperative discussions about intensive care admissions and severe complications.

Data presented as n (%) or mean (SD) unless otherwise specified. *adjusted for age, gender, ** adjusted for gender only due to collinearity with age; Abbreviations: **OR,** Odds Ratio; **SD**, standard deviation; **n**, number; **CPR** cardio-pulmonary resuscitation; **AD** advance directive; **CSD** Code Status Discussion. **Supplement 3. Patient factors and physicians’ preferences in perioperative discussions about intensive care admissions and severe complications**

| Factor | N | All | Discussion no | Discussion yes | Univariable OR (95% CI) | p-value | Multivariable OR (95% CI)* | p-value |
| --- | --- | --- | --- | --- | --- | --- | --- | --- |
| N |  | 467 | 104 | 363 |  |  |  |  |
| *Patients’ factors* | | | | | | | | |
| Advanced age (e.g. >80 years) yes, n (%) | 422 | 408 (96.7%) | 90 (94.7%) | 318 (97.2%) | 1.96 (0.64, 6) | 0.237 | 2.38 (0.75, 7.5) | 0.14 |
| Young age (e.g. < 50 years) yes, n (%) | 417 | 33 (7.9%) | 5 (5.3%) | 28 (8.7%) | 1.69 (0.63, 4.5) | 0.295 | 1.78 (0.66, 4.77) | 0.251 |
| Elective planned admission yes, n (%) | 418 | 64 (15.3%) | 15 (16.0%) | 49 (15.1%) | 0.94 (0.5, 1.76) | 0.843 | 0.98 (0.52, 1.85) | 0.958 |
| Inpatient status yes, n (%) | 420 | 134 (31.9%) | 25 (26.3%) | 109 (33.5%) | 1.41 (0.85, 2.36) | 0.185 | 1.55 (0.92, 2.62) | 0.098 |
| High expected perioperative complication risk yes, n (%) | 419 | 386 (92.1%) | 85 (92.4%) | 301 (92.0%) | 0.95 (0.4, 2.27) | 0.914 | 1.08 (0.44, 2.6) | 0.872 |
| Low expected perioperative complication risk yes, n (%) | 422 | 39 (9.2%) | 7 (7.4%) | 32 (9.8%) | 1.34 (0.57, 3.15) | 0.497 | 1.44 (0.61, 3.39) | 0.407 |
| Pre-existing complete need for care (e.g. nursing home) yes, n (%) | 420 | 363 (86.4%) | 73 (77.7%) | 290 (89.0%) | 2.32 (1.28, 4.21) | 0.006 | 2.38 (1.3, 4.35) | 0.005 |
| Low expected quality of life after surgery yes, mean (SD) | 419 | 379 (90.5%) | 80 (85.1%) | 299 (92.0%) | 2.01 (1, 4.03) | 0.049 | 2.16 (1.07, 4.38) | 0.032 |
| Significant comorbidities (e.g. advanced cardiac or pulmonary disease) yes, n (%) | 421 | 388 (92.2%) | 82 (87.2%) | 306 (93.6%) | 2.13 (1.01, 4.51) | 0.048 | 2.22 (1.04, 4.77) | 0.04 |
| High iatrogenic risk of circulatory arrest (heart surgery, intrathoracic procedures) yes, n (%) | 420 | 401 (95.5%) | 86 (91.5%) | 315 (96.6%) | 2.66 (1.04, 6.83) | 0.041 | 2.49 (0.96, 6.42) | 0.06 |
| Cognitive impairment or dementia yes, n (%) | 418 | 327 (78.2%) | 69 (73.4%) | 258 (79.6%) | 1.42 (0.83, 2.41) | 0.199 | 1.49 (0.87, 2.55) | 0.149 |
| Presence of a documented advance directive yes, n (%) | 419 | 330 (78.8%) | 72 (78.3%) | 258 (78.9%) | 1.04 (0.59, 1.82) | 0.895 | 1.03 (0.58, 1.81) | 0.92 |
| Known malignancy or hematologic cancer, n (%) | 421 | 294 (69.8%) | 61 (64.9%) | 233 (71.3%) | 1.34 (0.82, 2.18) | 0.237 | 1.34 (0.82, 2.19) | 0.247 |
| *Anaesthetists’ preferences* | | | | | | | | |
| After surgery, the patient's resuscitation status should be changed to the original resuscitation status yes, n (%) | 386 | 326 (84.5%) | 86 (93.5%) | 240 (81.6%) | 0.31 (0.13, 0.75) | 0.009 | 0.32 (0.13, 0.77) | 0.011 |
| In the case of perioperative resuscitation with subsequent ROSC, postoperative intensive care measures should be continued more intensively/aggressively, as the complication occurred in a surgical context yes, n (%) | 387 | 144 (37.2%) | 35 (38.0%) | 109 (36.9%) | 0.95 (0.59, 1.55) | 0.85 | 0.91 (0.56, 1.49) | 0.714 |
| The decision to discontinue therapy after perioperative resuscitation should involve both the surgical and anaesthesiology teams, with equal weighting of opinion yes, n (%) | 388 | 347 (89.4%) | 82 (90.1%) | 265 (89.2%) | 0.91 (0.42, 1.98) | 0.81 | 0.89 (0.4, 1.96) | 0.765 |
| When determining the perioperative resuscitation status, there are often interdisciplinary conflicts between surgery and anaesthesia yes, n (%) | 390 | 225 (57.7%) | 61 (66.3%) | 164 (55.0%) | 0.62 (0.38, 1.01) | 0.057 | 0.66 (0.4, 1.08) | 0.096 |
| An interdisciplinary determination/discussion of the resuscitation status between anaesthesia and surgery should be standard practice yes, n (%) | 387 | 351 (90.7%) | 82 (91.1%) | 269 (90.6%) | 0.94 (0.41, 2.14) | 0.878 | 0.95 (0.42, 2.18) | 0.906 |
| In view of the ageing population, perioperative resuscitation status should be discussed more frequently yes, n (%) | 387 | 375 (96.9%) | 86 (93.5%) | 289 (98.0%) | 3.36 (1.06, 10.69) | 0.04 | 4.07 (1.25, 13.26) | 0.02 |

**Supplementary Table 3.** Patient factors and physicians’ preferences in perioperative discussions about intensive care admissions and severe complications.

Data presented as n (%) or mean (SD) unless otherwise specified. *adjusted for age, gender. Abbreviations: **OR,** Odds Ratio; **SD**, standard deviation; **n**, number; **ROSC,** return of spontaneous circulation.

**Supplement 4.** CRF

**Survey of perioperative Code Status Anaesthesia**

**Survey on perioperative code status from the anaesthesiology perspective**

| **1. Introduction**  Dear Participant  Thank you for taking part in this survey on the discussion of perioperative code status. Your input is a valuable contribution toward understanding how code status is currently managed in the perioperative setting in Switzerland.  Please note the following information:   - This survey is intended for physicians who are actively working in the field of anaesthesiology. The questions specifically focus on the code status as it is discussed in the context of surgical procedures (perioperative care). - Discussions of code status related to non-surgical interventions (e.g. bronchoscopy, catheter angiography or stand-by anaesthesia) are not the focus of this survey. If you do not fall into the relevant category, please close this link. - The survey will take approximately 8 minutes to complete. It can be saved temporarily. - Please complete the survey only once.   Thank you for your participation.  Prof. Dr. med. Sabina Hunziker  Head of Medical Communication / Deputy Head of Psychosomatic Medicine, University Hospital Basel  Prof. Dr. med. Luzius Steiner  Chief Physician and Head of Anaesthesiology / Medical Director of the Department of Acute Medicine, University Hospital Basel  Prof. Dr. med. Thierry Girard  Chief Physician and Deputy Head of Anaesthesiology, University Hospital Basel |
| --- |

| **2. Personal Details** | | |
| --- | --- | --- |
| 2.1 | What is your age (in years)? | _________________________ |
| 2.2 | Your gender | - Female - Male - Prefer not to say |
| 2.3 | Where do you work? | - Tertiary Care Hospital (e.g. university hospital)/hospital with maximum care - Secondary Care Hospital - Regional Hospital (smaller Secondary Care Hospital) - Other |
| 2.4 | How many years of professional medical experience do you have? | _________________________ |
| 2.5 | What is your current position? | - Resident - Senior physician / Attending physician - Consultant - Chief physician |
| 2.6 | Do you hold a board-certified medical specialty? | - Yes - No |
| 2.7 | Which specialties are you board-certified in? | - Internal Medicine - Anaesthesiology - Intensive Care Medicine - Other |
| 2.8 | How many premedication consultations do you typically conduct per week? | _________________________ |
| 2.9 | On average, how many anaesthesia procedures do you perform per week? | _________________________ |
| 2.10 | How many perioperative resuscitations have you performed or witnessed? | - < 10 - 10-50 - > 50 |
| 2.11 | How many resuscitations (in any setting) have you performed or witnessed? | - < 10 - 10-50 - > 50 |

| **3. Perioperative Discussion of Code Status** | | |
| --- | --- | --- |
| **How often do you discuss perioperative code status in the following situations?** | | |
| 3.1 | Low surgical risk?  low = ASA 1-2, patients with minor illness and no functional limitation (e.g. well-controlled diabetes mellitus or hypertension); estimated 30-day mortality 0.02-0.14%. | - Almost always - Often - Rarely - Almost never |
| 3.2 | With an increased risk of surgery?  increased = ASA 3-4, patients with minor illness with functional limitation (e.g. COPD, heart failure); estimated 30-day mortality 1.4-11%. | - Almost always - Often - Rarely - Almost never |
| 3.3 | In the event of a life-threatening situation?  life-threatening = ASA 5, patient with life-threatening condition in which survival is not expected without surgery (e.g. ruptured abdominal aortic aneurysm, massive trauma); estimated perioperative mortality 50%. | - Almost always - Often - Rarely - Almost never |

| **Preoperative discussion of serious complications and intensive care measures**  **How often do you discuss serious complications in the following situations preoperatively?**  Serious complications = e.g. post-operative sepsis or serious bleeding complications requiring treatment in the intensive care unit. | | |
| --- | --- | --- |
| 3.4 | Low surgical risk?  low = ASA 1-2, patients with minor illness and no functional limitations (e.g. well-controlled diabetes mellitus or hypertension); estimated 30-day mortality 0.02-0.14%. | - Almost always - Often - Rarely - Almost never |
| 3.5 | With an increased risk of surgery?  increased = ASA 3-4, patients with minor illness with functional limitation (e.g. COPD, heart failure); estimated 30-day mortality 1.4-11%. | - Almost always - Often - Rarely - Almost never |
| 3.6 | In the event of a life-threatening situation?  life-threatening = ASA 5, patient with life-threatening condition in which survival is not expected without surgery (e.g. ruptured abdominal aortic aneurysm, massive trauma), perioperative mortality 50%. | - Almost always - Often - Rarely - Almost never |

| **Preoperative Discussion of Treatment Limitations**  **How often do you discuss limitations of postoperative treatment in the event of serious complications in the following situations?**  Postoperative treatment limitations = e.g. no prolonged intubation or avoidance of certain organ replacement therapies (e.g. hemodialysis). | | |
| --- | --- | --- |
| 3.7 | Low surgical risk?  low = ASA 1-2, patients with minor illness without functional limitations (e.g. well-controlled diabetes mellitus or hypertension); estimated 30-day mortality 0.02-0.14%. | - Almost always - Often - Rarely - Almost never |
| 3.8 | With an increased risk of surgery?  increased = ASA 3-4, patients with minor disease with functional limitations (e.g. COPD, heart failure); estimated 30-day mortality 1.4-11%. | - Almost always - Often - Rarely - Almost never |
| 3.9 | In the event of a life-threatening situation?  life-threatening = ASA 5, patient with life-threatening condition in which survival is not expected whithout surgery (e.g. ruptured abdominal aortic aneurysm, massive trauma), perioperative mortality 50%. | - Almost always - Often - Rarely - Almost never |

| **Discussion of the Code Status** | | |
| --- | --- | --- |
| 3.10 | When you talk to patients about the possibility of perioperative cardiac arrest: How should the perioperative code status be determined? | - I determine the code status, considering the patient's medical condition and preferences (= physician decision) - I discuss the perioperative code status with the patient and make a shared decision-(=shared decision making) - I inform the patient about perioperative resuscitation so that he/she can make his/her own decision (= patient decision) |
| 3.11 | In your opinion, who in the medical team is responsible for definitively determining the code status for the perioperative period? | - Anaesthetist - Both Anaesthetist and surgeon together - Surgeon |

| **In which of the following situations do you consider it necessary to discuss perioperative code status and potential intensive care measures?** | | | | | | | |  |
| --- | --- | --- | --- | --- | --- | --- | --- | --- |
|  |  | Not necessary | Often not necessary | Rarely necessary | Sometimes necessary | Often necessary | Always necessary | |
| 3.12 | Advanced age (e.g. >80 years) |  |  |  |  |  |  | |
| 3.12 | Young age (e.g. < 50 years) |  |  |  |  |  |  | |
| 3.12 | Elective admission |  |  |  |  |  |  | |
| 3.12 | Inpatient patient |  |  |  |  |  |  | |
| 3.12 | High expected risk of perioperative complications |  |  |  |  |  |  | |
| 3.12 | Low expected risk of perioperative complications |  |  |  |  |  |  | |
| 3.12 | Pre-existing complete dependency (e.g. nursing home) |  |  |  |  |  |  | |
| 3.12 | Low expected postoperative quality of life |  |  |  |  |  |  | |
| 3.12 | Significant comorbidities (e.g. severe heart or lung disease) |  |  |  |  |  |  | |
| 3.12 | High perioperative risk of cardiac arrest (cardiac surgery, intrathoracic procedure) |  |  |  |  |  |  | |
| 3.12 | Cognitive impairment or dementia |  |  |  |  |  |  | |
| 3.12 | Documented Advance Directives |  |  |  |  |  |  | |
| 3.12 | Advanced cancer or malignant hematologic disease |  |  |  |  |  |  | |

| 3.12 | What is your estimate of the average survival rate with good neurological outcome until hospital discharge after perioperative cardiac arrest and resuscitation until hospital discharge?  Good neurological outcome, CPC 1-2 = The patient can live independently. Certain neurological impairments (e.g. hemiplegia) may be present. | Your estimate: 0%-100%:  ___________ % |
| --- | --- | --- |

| 3.13 | Do you routinely ask patients whether they have an advance directive? | - Yes - Rather yes - Rather no - No |
| --- | --- | --- |
| 3.14 | In your opinion, Is the code status preference as documented in the advance directive also relevant for the perioperative phase? | - Yes - Rather yes - Rather no - No |
| 3.15 | Do you think that discussing perioperative code status before surgery causes patients to feel insecure or anxious? | - Yes - Rather yes - Rather no - No |
| 3.16 | Does your institution provide training on how to discuss code status with patients? | - Yes - No |
| 3.17 | In your opinion, what role does anaesthesiology play in discussing the perioperative code status, possible intensive care measures, and serious complications?  You may answer in keywords or brief statements: | ______________________ |

| **4. Pre-existing "Do Not Resuscitate " (DNR) Order.**  **The following questions address how to manage patients with documented DNR order prior to surgery (e.g. due to an advance directive or prior hospitalization).** | | |
| --- | --- | --- |
| 4.1 | Approximately what proportion you anesthetize have a pre-existing DNR order? | - Less than 25% - 25-50% - 50-75% - More than 75% |
| 4.2 | Do you address or re-discuss the perioperative code status with patients who have a pre-existing DNR order? | - Yes - Rather yes - Rather no - No |

| **To what extent do you agree with the following statements regarding perioperative management of patients with a DNR Order?** | | | | | | | |  |
| --- | --- | --- | --- | --- | --- | --- | --- | --- |
|  |  | Absolutely disagree | Disagree | Rather disagree | Tend to agree | Agree | Absolutely agree | |
| 4.3 | A "DNR order is illogical in the perioperative setting, as anaesthesia inherently involves resuscitation measures such as intubation and circulatory support. |  |  |  |  |  |  | |
| 4.3 | Anaesthesia and surgery affect the cardiopulmonary system; therefore, the medical team must be allowed to apply all necessary interventions, regardless of the patient's code status |  |  |  |  |  |  | |
| 4.3 | A DNR order must be respected perioperatively, as on resuscitation decisions should reflect the patient's values and not only the clinical judgment of the medical team. |  |  |  |  |  |  | |
| 4.3 | A DNR order should not be considered during surgery, as the  chances of successful resuscitation are higher in the monitored operating room environment. |  |  |  |  |  |  | |
| 4.3 | A DNR order is irrelevant perioperatively, since it is often difficult to distinguish between spontaneous and anaesthesia-induced cardiac arrest. |  |  |  |  |  |  | |
| 4.3 | Patients who are capable of consenting to a surgery and anaesthesia are also capable accepting or refusing perioperative resuscitation. |  |  |  |  |  |  | |

| 4.3 | In your opinion, how should anesthesiologists approach a documented DNR order in the perioperative setting?  You may answer in keywords or brief statements: | __________________________ |
| --- | --- | --- |

| **5. personal attitude towards periinterventional resuscitation status**  **To what extent do you agree with the following statements on perioperative and postoperative care?** | | | | | | | |  |
| --- | --- | --- | --- | --- | --- | --- | --- | --- |
|  |  | Absolutely disagree | Disagree | Rather disagree | Tend to agree | Agree | Absolutely agree | |
| 5.1 | After surgery, the patient's code status should revert to the original preoperative status. |  |  |  |  |  |  | |
| 5.1 | In the event of perioperative resuscitation with subsequent ROSC, postoperative intensive care should be continued more intensively/aggressively, as the complication occurred in a surgical context. |  |  |  |  |  |  | |
| 5.1 | The decision to discontinue therapy after perioperative resuscitation should involve both the surgical and anaesthesiology teams, with equal weighting of their opinions. |  |  |  |  |  |  | |
| 5.1 | There are often interdisciplinary conflicts between anaesthesia and surgery when determining the perioperative code status. |  |  |  |  |  |  | |
| 5.1 | An interdisciplinary discussion between anaesthesia and surgery regarding code status should be standard practice. |  |  |  |  |  |  | |
| 5.1 | Given the aging population, perioperative code status should be addressed more frequently. |  |  |  |  |  |  | |

| 5.2 | In your opinion, how should the perioperative code status ideally be determined and discussed?  You may answer in keywords or brief statements: | __________________________ |
| --- | --- | --- |
